# Recombinant Expression and Activity Analysis of a *Vibrio alginolyticus* Phage-Derived Endolysin LysV039C

**DOI:** 10.3390/cimb48080754

**Published:** 2026-07-25

**Authors:** Yiyuan Yu, Yingying Ye, Hongjiao Cai, Zhongqin Li, Jiang Zheng, Guixiang Tong, Mingfeng Xu, Qibiao Weng, Honglian Tan, Mao Lin

**Affiliations:** 1 Engineering Research Center of the Modern Technology for Eel Industry of Education Ministry, Fisheries College of Jimei University, Xiamen 361021, China; 202312951057@jmu.edu.cn (Y.Y.); 15659803796@163.com (Y.Y.); caihongjiao@jmu.edu.cn (H.C.); zhqinli@jmu.edu.cn (Z.L.); zhengjiang618@163.com (J.Z.); 2 Guangxi Key Laboratory of Aquatic Genetic Breeding and Healthy Aquaculture, Guangxi Academy of Fishery Sciences, Nanning 530021, China; tgx15@163.com (G.T.); hongliantan@163.com (H.T.); 3 Zhejiang Provincial Modern Biology and Medicine Industry College, Hangzhou Normal University (Cangqian Campus), Hangzhou 311231, China; zjxmf@163.com; 4 Key Laboratory of Eel Aquaculture and Processing of Fujian Province, Fuzhou 350200, China; 13338288037@189.cn

**Keywords:** bacteriophage, *Vibrio alginolyticus*, endolysin, recombinant expression

## Abstract

Pathogenic *Vibrio* species are a major cause of disease outbreaks in aquaculture, leading to substantial economic losses and posing risks to food safety. The increasing prevalence of antibiotic resistance among these pathogens has created an urgent need for alternative antimicrobial strategies. Phage-derived endolysins, which specifically degrade bacterial peptidoglycan, have emerged as promising candidates to replace or supplement conventional antibiotics. In this study, we systematically characterized the endolysin LysV039C from the *Vibrio alginolyticus* phage phiV039C. Whole-genome analysis identified a 462 bp endolysin gene, which was cloned into the pET-28a vector, expressed in E. coli BL21, and purified. The lytic activity of the recombinant enzyme was evaluated using a turbidity reduction assay. LysV039C showed the strongest activity (58.9%) against host bacteria in the logarithmic phase, with peak activity (60.7–64.1%) achieved at 53 μg/mL, 35 °C, and neutral pH. The addition of 2.5 mM EDTA enhanced activity to 63.8%, whereas 10 mM divalent metal ions strongly inhibited the enzyme (<6.5%). Lytic spectrum analysis demonstrated that LysV039C exhibited a broader lytic spectrum against six *Vibrio* species than its parent phage phiV039C. Overall, LysV039C combines high environmental adaptability (alkaline tolerance, suitability for aquaculture temperature ranges) with efficient and broadened lytic activity against multiple *Vibrio* species. These findings provide a foundation for developing eco-friendly agents for the prevention and control of pathogenic *Vibrio* and offer a valuable reference for the mining of other phage-derived antibacterials against aquatic pathogens.

## 1. Introduction

*Vibrio alginolyticus* belongs to the family *Vibrionaceae* and the genus *Vibrio*. As a typical Gram-negative pathogen, it can not only cause fish diseases such as gill rot disease [1] and red leg disease [2] but also lead to human symptoms such as diarrhea and septicemia [3,4], posing a potential risk to public health and safety. Currently, the primary means of preventing and treating *Vibrio* infections still relies on antibiotics [5,6]. However, the growing prevalence of antibiotic resistance and environmental contamination stemming from the widespread use of antimicrobial agents has become increasingly concerning [7,8,9]. Therefore, there is an urgent need to develop sustainable antibiotic alternatives to conventional antimicrobial therapy. Bacteriophage endolysins are bacteriophage-encoded peptidoglycan hydrolases that selectively disrupt the cell wall of pathogenic bacteria through precise cleavage of peptidoglycan bonds [10], offering advantages such as high efficiency, low residue, and a low tendency to induce resistance [11]. Furthermore, their activity can be enhanced through engineering modifications [12]. Recent studies demonstrate that *Vibrio*-specific phage endolysins exert potent and selective antibacterial activity against key aquaculture pathogens, including *Vibrio parahaemolyticus* and *Vibrio harveyi*. Functionally, characterized endolysins fall into three major classes—muramidases, endopeptidases, and amidases—each defined by its distinct catalytic specificity: muramidases cleave the β-1,4-glycosidic bonds in the peptidoglycan glycan backbone; endopeptidases hydrolyze peptide bonds within the stem peptides or cross-links; and amidases sever the amide bonds linking the glycan backbone to the peptide moiety [13].

Compared with Gram-positive phage endolysins—several of which have advanced to commercialization [14]—the practical application of Gram-negative phage endolysins still faces considerable challenges. First, the penetration efficiency of most endolysins into Gram-negative bacteria is hindered by the outer membrane barrier, rendering these bacteria largely insensitive to external lysins [15]. Second, environmental fluctuations, such as variations in temperature, salinity, and metal ion concentrations in aquaculture settings, can compromise the enzymatic activity and stability of lysins [16]. Research indicates that the chelating agent EDTA enhances lysin penetration by disrupting the lipopolysaccharide layer [17], thereby providing potential for applications against Gram-negative bacteria. Although some studies on *Vibrio* lysins exist [18,19], systematic investigation of *V. alginolyticus* phage lysins remains limited, with a lack of comprehensive analysis regarding their environmental adaptability and broad-spectrum efficacy. Despite these advances, several critical knowledge gaps remain unaddressed, particularly regarding phage-derived endolysins against *V. alginolyticus*. First, although a few *Vibrio* lysins have been reported, systematic data on their environmental adaptability—specifically the combined effects of temperature, pH, and metal ions that fluctuate markedly in aquaculture systems—are scarce. Such data are prerequisites for transitioning from in vitro efficacy to in situ application. Second, the outer membrane (OM) of *V. alginolyticus* poses a distinct permeability barrier, yet the optimal permeabilizer (EDTA) concentration required to synergize with its cognate endolysin has not been empirically defined, and simply extrapolating concentrations from other Gram-negative pathogens may lead to under- or over-dosing. Third, while the host range of *Vibrio* phages is often reported, a direct, quantitative comparison between the lytic spectrum of the parental phage and that of its recombinant endolysin is lacking; such a comparison is essential to evaluate whether the endolysin offers functional advantages beyond phage predation. Fourth, the physiological state of the target bacterium (e.g., growth phase) profoundly affects cell wall crosslinking and susceptibility to hydrolases, but this factor has not been systematically integrated into the activity assessment of Vibrio endolysins, leaving a gap in rational dosing strategies.

To bridge these gaps, this study isolated a novel phage (phiV039C) against *V. alginolyticus* and focused on its endolysin LysV039C. Given that phages and their endolysins are often assumed to share equivalent host ranges, we directly compared the lytic spectra of the phage particle and its recombinant endolysin to determine whether this assumption holds in the context of *V. alginolyticus*.

## 2. Materials and Methods

### 2.1. Bacterial Strains and Reagents

The following strains were maintained in our laboratory: *Vibrio alginolyticus* phage phiV039C and its host strain V039C; *Vibrio alginolyticus* strains V208, TY05, and TY12; *Vibrio parahaemolyticus* strains TY10, TY18, VP11, Vp2, Vp3, and GH32; *Vibrio vulnificus* TY13; *Vibrio tubiashii* TY45; *Aeromonas hydrophila* A009 and A015; *Bacillus amyloliquefaciens* TY27; *Streptococcus agalactiae* SA503; and *Staphylococcus aureus* YY05. Additionally, strains *Vibrio vulnificus* H1 and G1, *Edwardsiella* B79, and *Aeromonas hydrophila* B11 were kindly provided by the research group of Professor Guo Songlin. *Escherichia coli* BL21(DE3) was purchased from Nanjing Vazyme Biotech Co., Ltd. (Nanjing, China). LB broth and LB agar were obtained from Qingdao Hope Bio-technology Co., Ltd. (Qingdao, China). Agarose was sourced from Biowest (Nuaillé, France). The plasmid pET-28a(+), restriction enzymes BamHI and XhoI, 15% precast electrophoresis gels, and True Color dual-color pre-stained protein marker were purchased from Sangon Biotech (Shanghai) Co., Ltd. (Shanghai, China). Kanamycin hydrochloride was procured from Beijing Solarbio Science & Technology Co., Ltd. (Beijing, China). HisPur™ Ni-NTA Spin Columns were purchased from Thermo Fisher Scientific (Waltham, MA, USA). The BCA Protein Assay Kit was obtained from Abbkine Scientific Co., Ltd. (Wuhan, China).

### 2.2. Sequence Analysis of Endolysin LysV039C

The whole genome of phage phiV039C was sequenced using the PacBio SMRT [20] platform (GenBank accession number MN922296.1). Genome analysis revealed that ORF6 exhibits potential lytic activity and was designated LysV039C. Subsequently, we used SnapGene (6.0.2) software to translate the nucleotide sequence of the lysin and analyze its physicochemical properties, including molecular weight, amino acid composition, and signal peptides. Protein sequences homologous to LysV039C were retrieved and downloaded from the NCBI database via the BLAST (WebBLAST). Sequence alignment was conducted with the ClustalW function in MEGA software (MEGA 11) [21], and a phylogenetic tree was constructed using the Neighbor-Joining method [22]. Finally, the conserved domains of the lysin were predicted using the CD-Search tool on the NCBI website (https://www.ncbi.nlm.nih.gov/Structure/cdd/wrpsb.cgi, accessed on 23 July 2026).

### 2.3. Heterologous Expression of Endolysin LysV039C

Primers LysV039C-F1 (5′-CGCGGATCCATGGACCCTAAATACATAAC-3′) and LysV039C-R1 (5′-CCGCTCGAGTCAATCATTGCCCCACT-3′) were designed based on the lysin gene of phiV039C, with a BamHI site at the 5′ end of F1 and an XhoI site at the 5′ end of R1. After PCR amplification, the products were analyzed for fragment size using 1% agarose gel electrophoresis. The restriction-digested PCR product was ligated with the plasmid pET-28a(+) using T4 DNA ligase [23], and the resulting construct was transformed into competent *E. coli* BL21 cells, yielding the recombinant expression strain BL21-pET-LysV039C.

BL21-pET-LysV039C was streaked onto LB solid medium and incubated at 37 °C for 12 h. Single colonies were selected from the plate and inoculated into 10 mL of LB broth containing 50 μg/mL kanamycin (Kana). The culture was shaken at 37 °C and 180 rpm for 12 h. Then, 1 mL of this culture was transferred to 100 mL of fresh LB broth containing 50 μg/mL Kana and incubated under the same conditions until the OD_600_ reached 0.60 ± 0.05. Isopropyl β-D-1-thiogalactopyranoside (IPTG) was then added to a final concentration of 1 mM, and induction continued at 37 °C, 180 rpm for 8 h. After induction, the culture was centrifuged at 4 °C and 10,000 rpm for 5 min. The supernatant was discarded, and the pellet was resuspended in 10 mL of ice-cold PBS. This washing procedure was repeated three times. The resuspended cells were lysed by ice-bath ultrasonication (90 W, 1 s on/3 s off for 5 min). The lysate was centrifuged at 10,000 rpm for 5 min, and the supernatant containing the lysin was collected. The expression pattern of the recombinant protein was assessed by SDS-PAGE.

### 2.4. Purification and Concentration Determination of Endolysin LysV039C

After cell disruption, the supernatant was filtered through a 0.22-μm membrane and gently loaded onto a Ni-NTA nickel-affinity column for purification of His-tagged recombinant protein [24]. Bound proteins were eluted sequentially with 6 mL of imidazole elution buffer at each of the following concentrations: 10, 20, 50, 100, 200, and 300 mmol/L. Following buffer exchange with an Amicon Ultra-15 10 kDa (Sigma-Aldrich, Shanghai, China)centrifugal filter device, the purified LysV039C was supplemented with glycerol at a final concentration of 20% (*v*/*v*) as a cryoprotectant and subsequently stored at −80 °C. The purified LysV039C was analyzed by SDS-PAGE, and the concentration of the recombinant protein was determined using a BCA protein assay kit (Yakun Biotechnology Co., Ltd., Wuhan, China).

### 2.5. Determination of the Bacteriolytic Activity of Endolysin LysV039C

The bactericidal activity of recombinant endolysin LysV039C was evaluated using a turbidity reduction assay [25]. *V. alginolyticus* V039C was grown to the logarithmic phase, then harvested by centrifugation (8000 rpm, 4 °C, 2 min), and the supernatant was discarded. The pellet was resuspended in PBS buffer (pH 7.4), and this washing procedure was repeated three times. The bacterial suspension was then adjusted to an OD_600_ of 2.0 ± 0.1. In a 96-well plate, 100 μL of the bacterial suspension was mixed with 100 μL of the ultrasonication supernatant from the recombinant strain BL21-pET-LysV039C. PBS (pH 7.4) served as the negative control, and commercial endolysin (final concentration 20,000 U/mg) was used as the positive control. Each treatment was performed in triplicate. After incubation with shaking at 30 °C for 2 h, the OD_600_ was measured using a microplate reader. The turbidity reduction rate (%) was calculated as follows:

(1) $$(\mathrm{initial}\;{\mathrm{OD}}_{600}-\mathrm{final}\;{\mathrm{OD}}_{600})/\mathrm{initial}\;{\mathrm{OD}}_{600}$$

### 2.6. Optimal Conditions for Endolysin LysV039C Activity

#### 2.6.1. Bactericidal Activity of Endolysin LysV039C Against Bacteria at Different Growth Phases

Cultures of *V. alginolyticus* V039C were harvested at 3 h (lag phase), 12 h (logarithmic phase), and 24 h (stationary phase). The cultures were centrifuged at 4 °C and 8000 rpm for 2 min and resuspended in PBS (pH 7.4) to an OD_600_ of 2.00 ± 0.10. In a 96-well plate, 100 μL of the bacterial suspension was combined with 100 μL of a solution containing LysV039C (final concentration 51 μg/mL) and EDTA (final concentration 0.5 mmol/L). PBS (pH 7.4) served as the negative control. During a 2 h incubation at 30 °C, the OD_600_ was measured every 10 min with a microplate reader.

#### 2.6.2. Optimal Concentration of Endolysin LysV039C and Synergistic Effect with EDTA

A suspension of *V. alginolyticus* V039C was prepared by culturing to log phase (OD_600_ of 1.10 ± 0.05), centrifuging at 8000 rpm for 2 min at 4 °C, and resuspending in PBS (pH 7.4) to a final OD_600_ of 2.00 ± 0.10. In a 96-well plate, 100 μL of this suspension was mixed with 100 μL of a solution containing LysV039C (final concentrations: 0, 8.5, 17, 35, 51, 68, 85, and 102 μg/mL) and EDTA (final concentration 0.5 mmol/L). Separately, 100 μL of the same bacterial suspension was mixed with 100 μL of a solution containing LysV039C (51 μg/mL) and EDTA at varying final concentrations (0, 0.5, 1.25, 2.5, 5, 10 mmol/L). PBS (pH 7.4) served as the blank control for all groups, with three replicates per condition. OD_600_ was measured after incubation.

### 2.7. Effects of Different Physicochemical Factors on Endolysin LysV039C

In a 96-well plate, 100 μL of the *V. alginolyticus* V039C suspension (cells harvested at logarithmic phase and resuspended to an OD_600_ of 2.0 ± 0.1) was mixed with LysV039C (final concentration 53 μg/mL) and EDTA (final concentration 0.5 mmol/L). Then, an equal volume of salt solution (i.e., 100 μL) was added to each well. The final concentrations of ZnCl_2_, CaCl_2_, MgCl_2_, MnCl_2_, and FeSO_4_ in the reaction system were each adjusted to 0.1, 1, and 10 mmol/L (separately).

A 3 mL aliquot of the *V. alginolyticus* V039C suspension (cells harvested at logarithmic phase and resuspended to an OD_600_ of 2.0 ± 0.1) was mixed with LysV039C (final concentration 51 μg/mL) and EDTA (final concentration 0.5 mmol/L) in a 10 mL centrifuge tube. The mixture was incubated at temperatures ranging from 10 to 45 °C for 2 h, after which the OD_600_ was measured.

*V. alginolyticus* V039C (OD_600_ = 1.10 ± 0.05) was harvested by centrifugation at 8000 rpm for 2 min at 4 °C. The supernatant was discarded, and the pellet was resuspended in PBS (pH 7.4); this washing step was repeated three times. For the final resuspension, the cell pellet was resuspended in equal volumes of PBS adjusted to pH 3, 4, 5, 6, 7, 8, 9, or 10, yielding bacterial suspensions with an OD_600_ of 2.0 ± 0.1. In a 96-well plate, 100 μL of each pH-adjusted suspension was mixed with LysV039C (final concentration 51 μg/mL) and EDTA (final concentration 0.5 mmol/L). After incubation at 30 °C for 2 h, OD_600_ was measured. Parallel controls containing PBS (at matching pH values) instead of endolysin were included. The turbidity reduction rate was calculated as (1). All conditions were tested in triplicate.

### 2.8. Lysis Spectrum Detection of Endolysin LysV039C and Phage phiV039C

Using the method described in Section 2.5, host bacterial cultures were processed to obtain resuspended cells with an OD_600_ of 2.00 ± 0.10 [26]. In a 96-well plate, 100 μL of bacterial suspension was sequentially added to 100 μL of a mixture containing endolysin LysV039C (final concentration 51 μg/mL) and EDTA (final concentration 0.5 mmol/L). After incubation at 30 °C for 2 h, the OD_600_ value was measured using a microplate reader.

## 3. Results

### 3.1. Sequence Analysis and Prediction of Endolysin LysV039C

**Figure 1:**
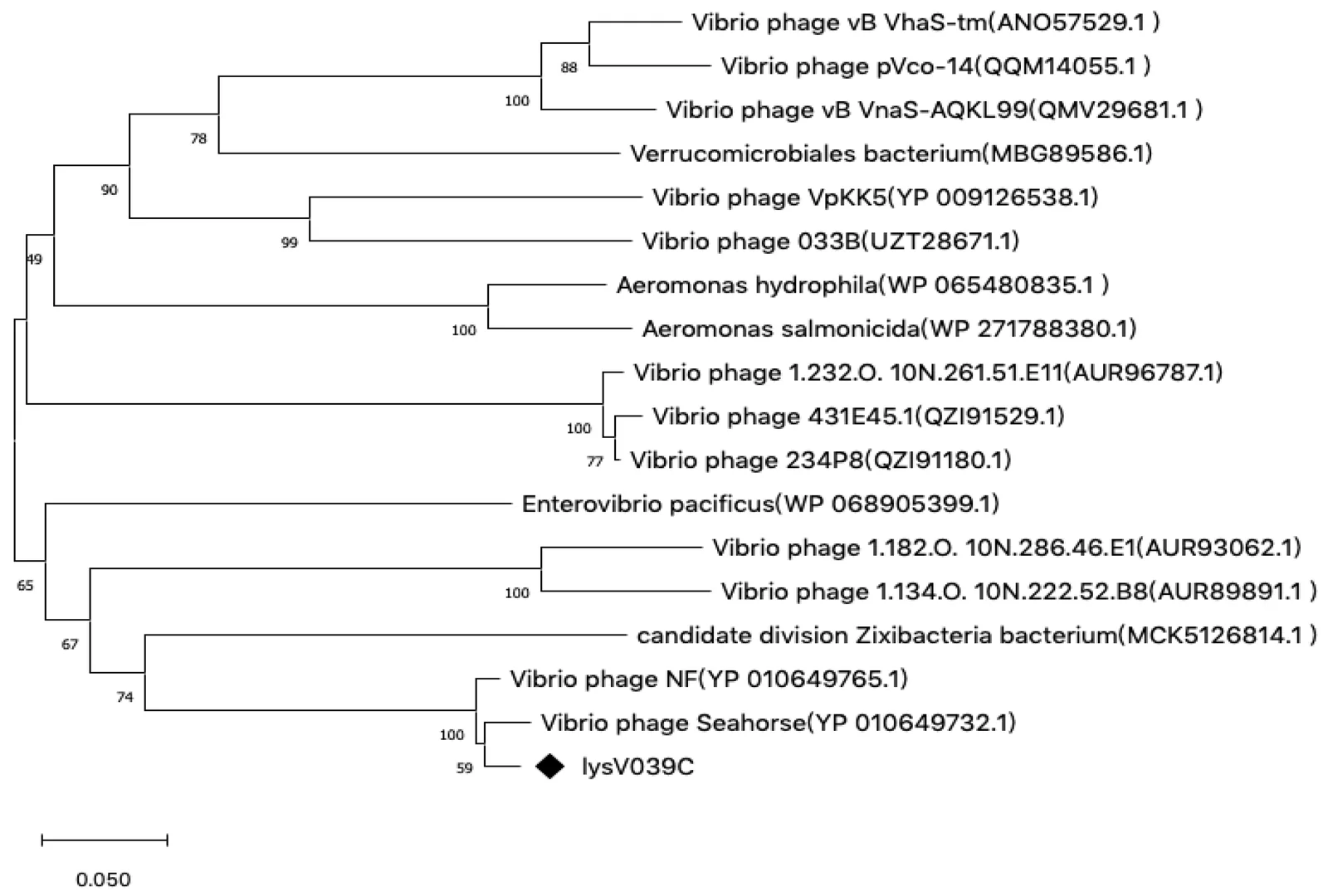
The phylogenetic tree of phage based on LysV039C.

The endolysin gene LysV039C (GenBank accession number QJD54561.1) from phage phiV039C was translated into an amino acid sequence using SnapGene for physicochemical analysis. The gene sequence comprises 462 bp, encoding a protein of 153 amino acids with a theoretical molecular weight of approximately 17.3 kDa. Bioinformatic analysis predicted that LysV039C lacks a signal peptide sequence. LysV039C showed high sequence overlap with a fragment of a *Vibrio* phage NF gene (Figure 1). It also clustered on the same phylogenetic branch as this NF gene. These results are consistent with the whole-genome comparison and indicate a close phylogenetic relationship between phages phiV039C and NF. CD-search prediction revealed that endolysin LysV039C contains an endolysin domain (PHA00447) spanning amino acids 4–148, a peptidoglycan-binding domain (PGRP, cd06583) spanning amino acids 2–121 (Table 1), and all other predicted domains are associated with amidase activity. All of these domains are catalytic domains characteristic of lysins, and no independent, non-catalytic cell-wall binding domains (such as SH3b or LysM) were detected, except for the PGRP domain which also possesses amidase activity.

### 3.2. Cloning, Expression, Purification, and Activity Determination of Endolysin LysV039C

**Figure 2:**
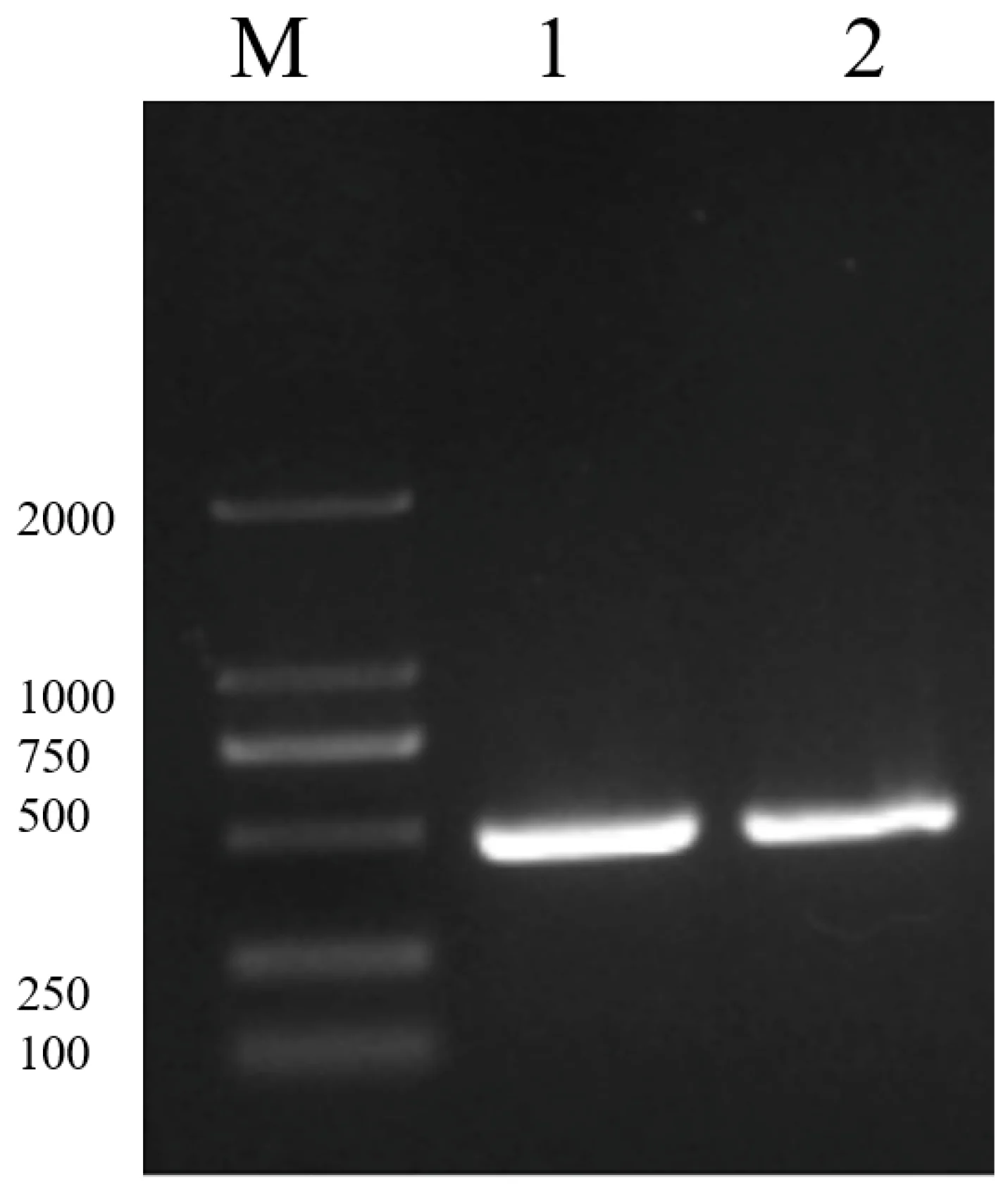
The PCR amplification products of LysV039C. Figure legend—Lane M: DNA marker. Lane 1, 2: LysV039C.

**Figure 3:**
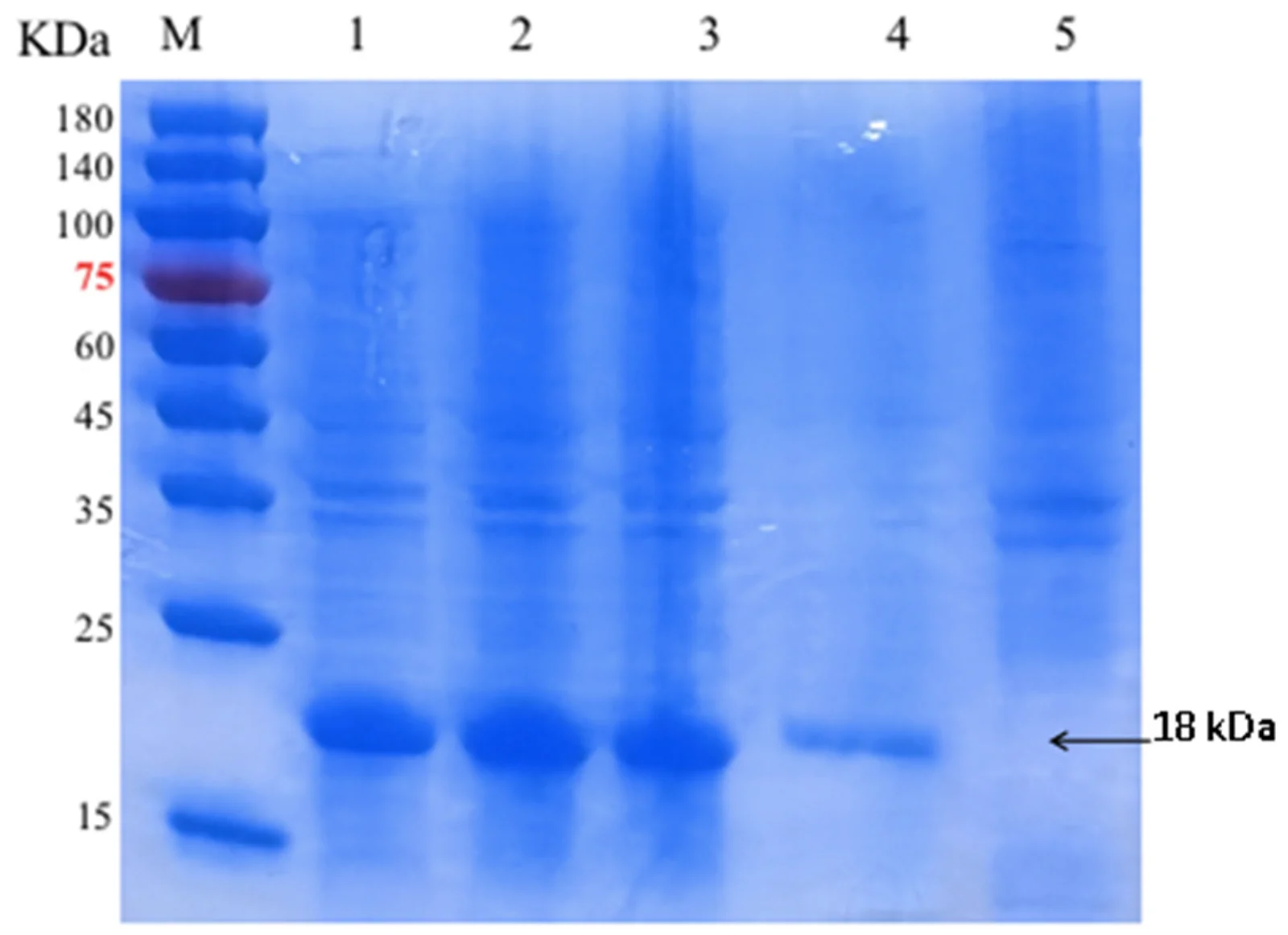
The expression of LysV039C in BL21-pET-LysV039C. Figure legend—Lane M: Protein marker. Lane 1: BL21-pET28a(+). Lane 2: Unpurified supernatant. Lanes 3, 4: First and second eluates from the nickel column. Lane 5: The total cell protein of BL21-pET28a(+).

PCR amplification was performed using the phage phiV039C genome as a template with primers LysV039C-F1 and LysV039C-R1. Gel electrophoresis analysis of the PCR product revealed a band of approximately 500 bp, consistent with the expected size of 462 bp (Figure 2). The target fragment was double-digested and then ligated into the pET-28a(+) plasmid. The recombinant plasmid was then transformed into E. coli BL21 competent cells. After sequence verification, large-scale soluble expression of LysV039C was induced with IPTG. Following ultrasonication and disruption of the cells, LysV039C was recovered in the supernatant. The purified protein showed a band at ~18 kDa by SDS-PAGE, aligning with the theoretical molecular weight (Figure 3). The concentration of recombinant LysV039C, quantified by the BCA assay, was 159 μg/mL.

**Figure 4:**
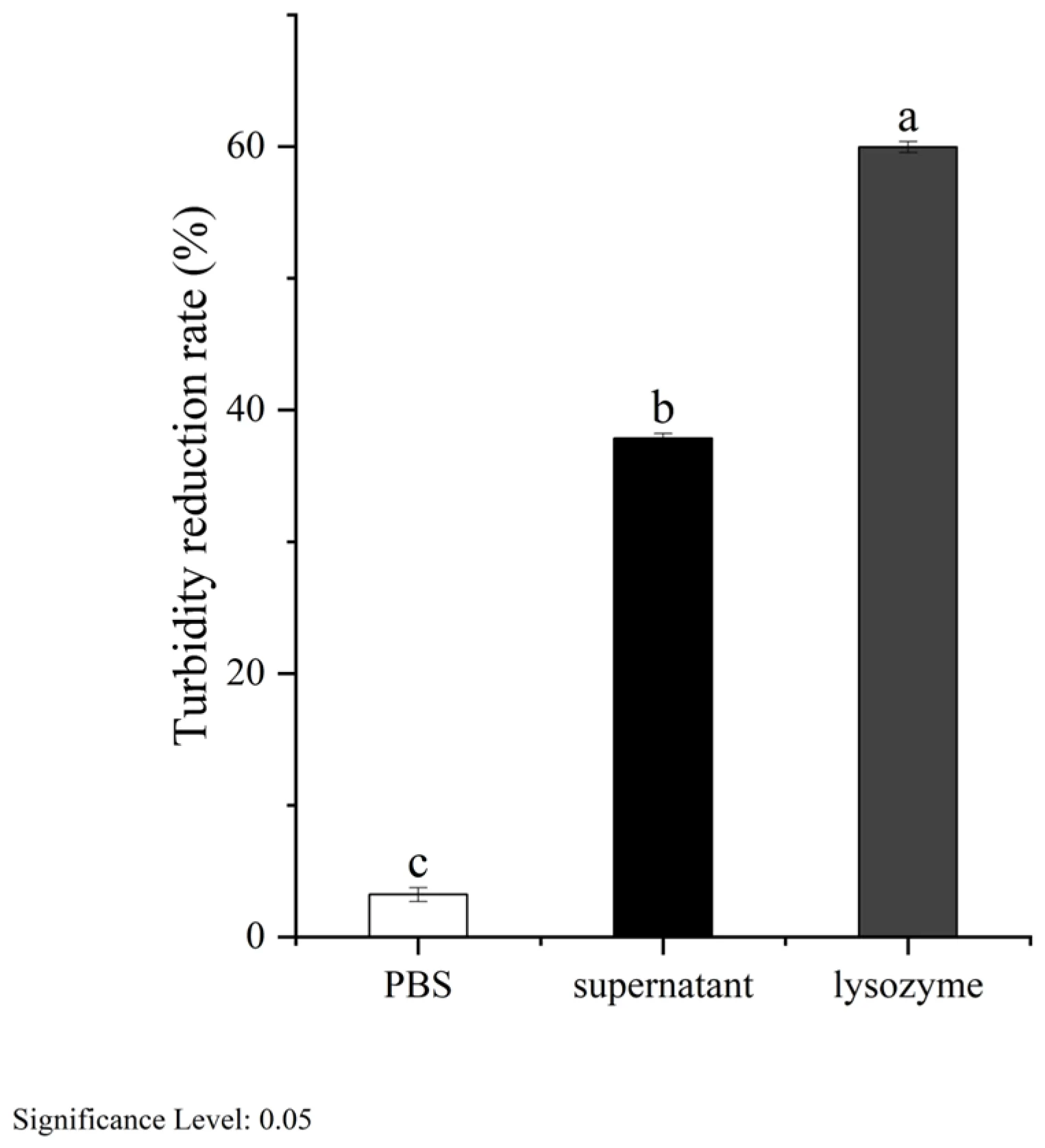
Bactericidal activity of the supernatants of BL21-PET-Lys against host bacteria. Different lowercase letters denote significant differences between groups (*p* < 0.05).

Bacterial concentration is directly proportional to the optical density at 600 nm (OD_600_) [27]. Intact bacterial cells have a high OD600, while endolysis causes a decrease. *V. alginolyticus* V039C was treated with the ultrasonication supernatant from the recombinant expression strain. The resulting turbidity reduction rate was 44.8% (Figure 4). In the PBS negative control, turbidity reduction was minimal (<5%), confirming the bactericidal activity of the recombinant supernatant.

### 3.3. Optimal Conditions for Endolysin LysV039C Activity

**Figure 5:**
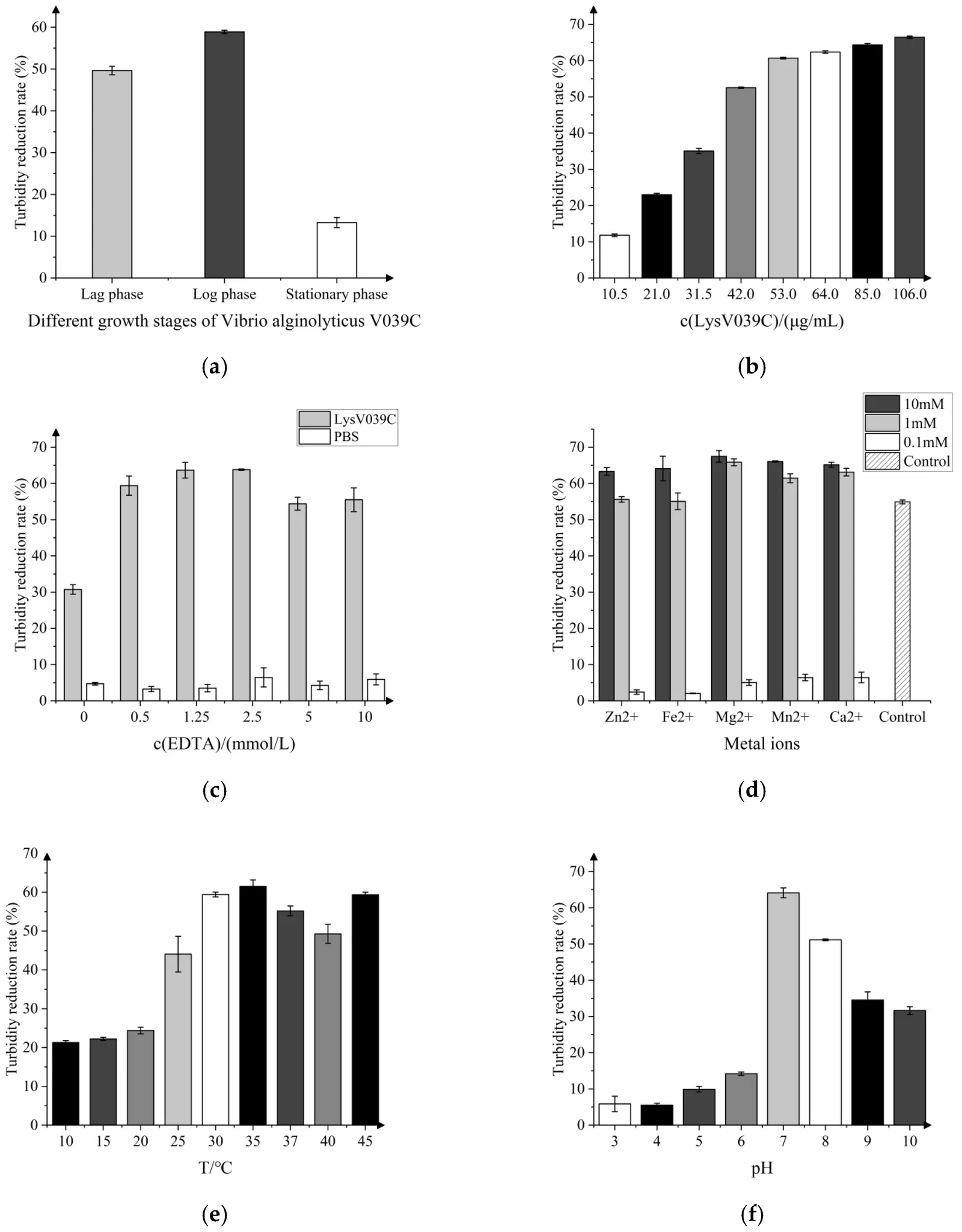
Factors affecting the bacteriolytic activity of the recombinant endolysin LysV039C. (**a**): Different growth phases of the host, lag phase (OD_600_ < 0.17), log phase (OD_600_ 0.17–1.10), and stationary phase (OD_600_ > 1.10). (**b**): Final concentration of LysV039C. (**c**): Final concentration of EDTA. (**d**): Final concentration of metal ions. (**e**): Temperature. (**f**): pH.

Endolysin LysV039C demonstrated distinct lytic effects on host bacteria across different growth stages (Figure 5a). The lytic activity against bacteria in the lag phase and stationary phase was 42.4% and 10.3%, respectively. For bacteria in the logarithmic growth phase, the lytic activity of recombinant LysV039C reached 58.9%, while the lag and stationary phases suppressed enzyme activity. At a concentration of 42 μg/mL, endolysin LysV039C showed a lytic activity of 52.5%. When the concentration was increased to 53 μg/mL, lytic activity reached 60.7%. However, when the concentration was further increased to 106 μg/mL, the bactericidal activity increased by only 9.5 percentage points, indicating that 53 μg/mL is the optimal bactericidal concentration for recombinant endolysin LysV039C (Figure 5b). With the synergistic action of 2.5 mM EDTA, the lytic activity of recombinant LysV039C increased significantly from 24.2% to 63.8% (Figure 5c). EDTA alone had no bactericidal effect but markedly enhanced the lytic activity of the lysin, as confirmed by negative controls. Figure 5d illustrates the effects of divalent metal ions on the lytic activity of recombinant LysV039C. At concentrations of 0.1 mM and 1 mM, Zn^2+^, Fe^2+^, Mg^2+^, Mn^2+^, and Ca^2+^ slightly enhanced the lytic activity. However, at 10 mM, the activity decreased to below 6.5%, indicating that high concentrations of divalent metal ions significantly inhibited lysin function. Evaluation of thermal stability (Figure 5e) revealed that lytic activity remained below 25% at temperatures between 10 and 20 °C. As the temperature increased to 35 °C, the lytic activity peaked at 61.5%. This is consistent with the optimal range for mesophilic enzymes and is suitable for most aquaculture environments. The influence of pH on lytic activity is presented in Figure 5f. With increasing pH, activity initially rose and then declined, reaching a maximum of 64.1% at pH 7. Even at pH 10, activity remained above 30%, indicating an optimal pH of 7 and demonstrating tolerance to alkaline conditions.

In summary, the lytic activity of endolysin LysV039C was significantly influenced by the host bacterial growth phase, enzyme concentration, synergy with EDTA, and environmental factors (temperature, pH, and divalent metal ions). Optimal activity was observed under the following conditions: logarithmic growth phase bacteria (58.9% lysis), lysin concentration of 53 μg/mL (60.7% lysis), temperature of 35 °C (61.5% lysis), pH 7 (64.1% lysis), and 2.5 mM EDTA (63.8% lysis). Notably, high concentrations (10 mM) of divalent metal ions strongly inhibited enzyme activity.

### 3.4. Lysis Spectrum of Endolysin LysV039C and Phage phiV039C

**Figure 6:**
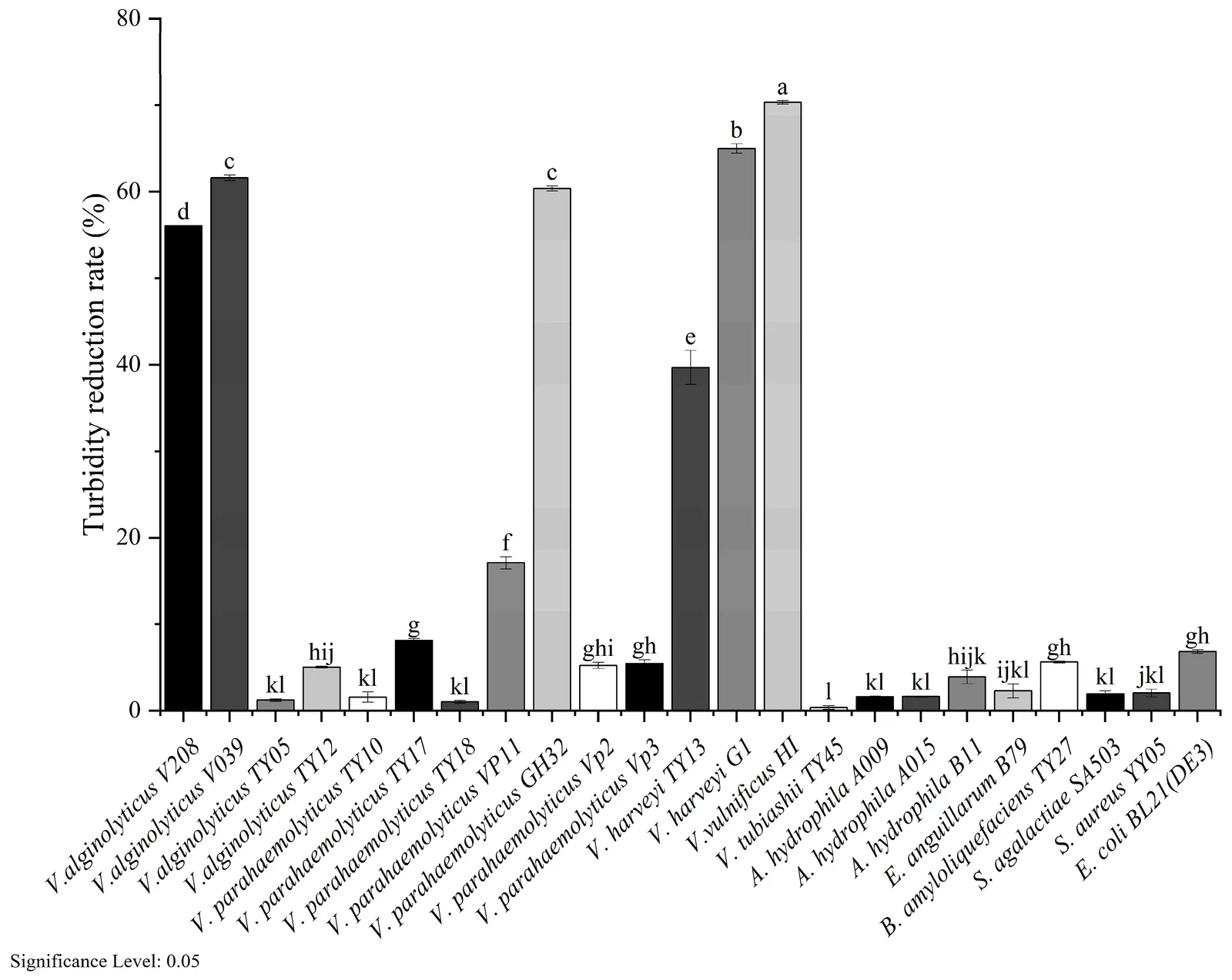
The bacteriolytic activity of the recombinant endolysin LysV039C against different bacteria. Different lowercase letters denote significant differences between groups (*p* < 0.05).

Endolysin LysV039C exhibited a broad lysis spectrum against Gram-negative bacteria beyond its host strain *V. alginolyticus* V039C, including *V. alginolyticus* V208 (56.1%), *V. vulnificus* H1 (70.4%), *V. parahaemolyticus* VP11 (17.1%), *V. parahaemolyticus* GH32 (60.4%), *V. vulnificus* TY13 (39.7%), and *V. harveyi* G1 (65.0%) (Figure 6). In contrast, lytic activity against Gram-positive bacteria—*S. aureus* YY05 (2.1%), *S. agalactiae* SA503 (1.9%), and *B. amyloliquefaciens* TY27 (5.6%)—was negligible. A spot assay confirmed that the parent phage phiV039C lysed only its host V039C, indicating that the recombinant lysin has a broader lytic spectrum than its parental phage. Among all 15 tested strains, the parent phage phiV039C, as confirmed by spot assay, produced clear plaques exclusively on its original host V039C. In contrast, recombinant LysV039C exhibited detectable lytic activity against 6 out of 6 Vibrio species tested, including non-host species such as *V. vulnificus* and *V. harveyi*, representing a relative expansion in spectrum coverage compared with its parental phage. Notably, the endolysin showed negligible activity against Gram-positive species, confirming its specificity for Gram-negative peptidoglycan architecture.

## 4. Discussion

A recombinant *E. coli* BL21(DE3) strain was established to efficiently express the phage-derived endolysin LysV039C. The LysV039C gene was cloned into a prokaryotic expression vector. The resulting recombinant plasmid was then transformed into *E. coli* BL21(DE3) competent cells to achieve heterologous expression. Sequence analysis revealed high sequence similarity with the lysin from *Vibrio* phage NF and confirmed the presence of an endolysin catalytic domain and a PGRP domain, which functions as a bifunctional module with both peptidoglycan recognition and amidase activity. This domain architecture is consistent with the functional characteristics of amidase-class lysins [28]. Compared with other Vibrio phage endolysins such as LysVPB [29], which possesses a putative calcium-binding site, LysV039C features a more compact PGRP-based design that integrates binding and catalytic functions within a single domain. The recombinant endolysin LysV039C accumulated predominantly in soluble form within the cytoplasm, minimizing inclusion body formation [30]. Following purification by nickel affinity chromatography, a high-purity LysV039C protein was obtained at a concentration of 159 μg/mL.

Endolysins naturally rely on cognate holins to permeabilize the cytoplasmic membrane and access periplasmic peptidoglycan during phage infection [31,32,33]. A subset of lysins can also achieve membrane translocation via N-terminal signal peptides independent of holins [34,35]. Bioinformatic analysis confirmed that LysV039C lacks a canonical N-terminal signal peptide, and our heterologous expression system did not co-express its native holin. This explains why recombinant LysV039C accumulated in the *E. coli* cytoplasm without triggering bacterial autolysis, consistent with the typical behavior of Gram-negative phage endolysins in prokaryotic expression systems.

As a macromolecular peptidoglycan hydrolase, exogenous LysV039C cannot freely penetrate the Gram-negative outer membrane barrier [36], which accounts for its moderate baseline lytic activity (24.2%) against intact *V. alginolyticus* cells. The chelating agent EDTA disrupts outer membrane stability by sequestering structural divalent cations (Mg^2+^, Ca^2+^) [37], and we found that supplementation with 2.5 mM EDTA enhanced LysV039C activity by 1.6-fold, reaching a lysis rate of 63.8%. Notably, the optimal EDTA concentration for LysV039C is slightly higher than the 0.5–1 mM typically reported for endolysins targeting other Gram-negative pathogens, such as Lys66 against *Proteus vulgaris* [38] and LysTAC1 against *Acinetobacter baumannii* [39]. This discrepancy likely reflects species-specific differences in lipopolysaccharide composition and outer membrane rigidity of *V. alginolyticus*, suggesting that permeabilizer optimization should be carefully evaluated when designing endolysin-based applications for specific aquaculture pathogens.

The lytic activity of phage lysins is strongly influenced by the physiological state of host bacteria, with the growth phase being a key determinant. This study demonstrated that the antibacterial efficacy of recombinant endolysin LysV039C was closely associated with bacterial growth stage. Maximum lytic activity against the host strain *V. alginolyticus* V039C was observed during the logarithmic growth phase, whereas activity decreased significantly against stationary-phase cells. Specifically, LysV039C exhibited lytic activity against logarithmic-phase bacteria that was 1.95 times higher than that against stationary-phase organisms. This growth-phase-dependent activity pattern is shared by other lysins, such as phage lysin AB54, which also showed superior activity against logarithmic-phase *Acinetobacter baumannii* [40]. The underlying mechanism involves substantial differences in cell wall structure, composition, and metabolic activity across growth phases [41]. Targeting pathogens during their most vulnerable growth stage may therefore optimize lysin-mediated bactericidal outcomes.

Recombinant endolysin LysV039C demonstrated favorable adaptability to temperature and pH. It showed optimum activity at 35 °C and pH 7.0, while maintaining relatively high activity at 25–37 °C and pH 7.0–9.0, conditions compatible with most marine and freshwater aquaculture environments. This temperature/pH profile is comparable to that of LysVpKK5 (optimal at 30 °C) [42], yet LysV039C retains >30% activity even at pH 10, whereas LysVpKK5 shows no activity at pH ≤ 7. In contrast, LysV039C maintains >30% activity at pH 10 while also retaining robust activity across pH 6–9. This difference in pH profiles suggests that marine *vibrio* phage endolysins may vary considerably in their pH preference. Within the concentration range of 0.1 to 1.0 mM, divalent metal ions moderately enhanced LysV039C lytic activity. In contrast, high concentrations (10 mM) strongly inhibited activity (<6.5% remaining), a phenomenon also reported for endolysin PlyD4 [43] and peptidoglycan hydrolase M15A [44]. This inhibition may be attributed to metal ion-induced conformational changes in the enzyme or to competitive binding with the substrate. This highlights the importance of regulating metal ion concentrations in practical applications. When considered alongside the metal ion inhibition profiles and EDTA synergy concentrations determined here, these data provide an initial reference for predicting how LysV039C might behave under the fluctuating physicochemical conditions typical of aquaculture systems—parameters that cannot be deduced from sequence analysis or phage host-range data alone.

Recombinant lysin LysV039C exhibited efficient lytic activity not only against its original host *V. alginolyticus* but also against various other Gram-negative pathogens, including *V. vulnificus*, *V. harveyi*, and *E. coli*. This cross-species lytic activity suggests a potential advantage in scenarios involving mixed infections, although this remains to be validated in complex biological systems such as host-associated microbiota or infected tissues. Although the antibacterial spectrum of LysV039C is limited and influenced by variations in outer membrane structure across bacterial species, it is markedly broader than that of its parent phage. A similar phenomenon has been documented for LysVPp1, whose lytic spectrum against *Vibrio* species was also broader than that of its parent phage CH20 [45]. While the limited number of reported cases precludes a definitive conclusion that this phenomenon is universal among *Vibrio* phages, the parallel observations in LysV039C and LysVPp1 suggest that the functional divergence between a phage particle and its encoded endolysin may be a recurring theme in at least some *Vibrio* phage systems. This underscores the importance of characterizing endolysins directly, as their host range cannot be reliably inferred from that of the parent phage.

However, this experiment still has certain stability issues. First, all lytic activity data were obtained in vitro using PBS-buffered bacterial suspensions; we have not tested LysV039C in live fish or shrimp challenge models, so its therapeutic efficacy, dosing regimen, and clearance in aquaculture animals remain unknown. Second, our assays used planktonic cells exclusively; biofilm eradication was not examined, and given that *vibrios* readily form biofilms on tank surfaces and fish mucus, this represents a major knowledge gap. Third, the antibacterial activity was evaluated solely by the turbidity reduction assay, which measures the decrease in optical density as an indirect indicator of cell lysis. This method is inherently semi-quantitative and can be confounded by cell aggregation, changes in cell morphology, or residual debris, and it does not directly measure viable cell counts. A more rigorous log-reduction assay (CFU/mL enumeration) is needed to accurately determine the bactericidal efficacy and killing kinetics of LysV039C. Fourth, our optimization of reaction conditions (Figure 5b–f) was performed solely with logarithmic-phase cells; stationary-phase cells, which are likely predominant in infected hosts, were not tested, so the optimal parameters for physiologically relevant bacterial populations remain to be established.

In summary, by targeting conserved structural motifs in the peptidoglycan layer, this recombinant lysin achieves effective cross-species lysis—offering a promising basis for developing novel antimicrobial agents against Gram-negative pathogens.

## 5. Conclusions

This study reveals that the biochemical properties and environmental adaptability of the phage-derived endolysin LysV039C, rather than its parent phage host range, define its potential for controlling multiple *Vibrio* species, with a lytic spectrum significantly broader than that of its parent phage. Despite being isolated from a single *Vibrio alginolyticus* phage with narrow host specificity, the recombinant endolysin exhibited potent lytic activity across six *Vibrio* species, demonstrating a markedly wider lytic spectrum than its parent phage phiV039C. Regarding the enzyme’s functional properties, the chelating agent EDTA exerted a critical synergistic role in disrupting the outer membrane barrier of Gram-negative bacteria, while divalent metal ions at aquaculture-relevant concentrations strongly inhibited enzymatic activity. These findings highlight the buffering and sensitizing capacity of outer membrane permeabilizers in overcoming the intrinsic resistance of Gram-negative pathogens to exogenous lysins. The optimal activity of LysV039C at 25~37 °C and neutral to alkaline pH, conditions compatible with most aquaculture environments, suggests that under well-managed application protocols, this endolysin could serve as an effective eco-friendly antimicrobial agent. However, its practical application as an eco-friendly antimicrobial agent will require further evaluation in live animal models, biofilm environments, and with direct bacterial enumeration methods. The stark contrast between the narrow host range of phage phiV039C and the broad lytic spectrum of its recombinant endolysin LysV039C provides fundamental evidence for understanding the functional divergence between phage particles and their encoded lytic enzymes, particularly in the context of developing novel biocontrol strategies against pathogenic Vibrio in aquaculture.

## Figures and Tables

**Table 1:** Conserved domain prediction for endolysin LysV039C.

Name	Accession	Description	Interval	E-Value
PHA00447	PHA00447	Lysozyme	4–148	1.67 × 10^−44^
PGRP	cd06583	Peptidoglycan recognition proteins (PGRPs) are pattern recognition receptors that bind.	3–121	1.02 × 10^−23^
Ami_2	smart00644	Ami_2 domain	2–119	5.63 × 10^−14^
AmpD	COG3023	N-acetyl-anhydromuramyl-L-alanine amidase AmpD	1–121	1.02 × 10^−9^
Amidase_2	pfam01510	N-acetylmuramoyl-L-alanine amidase	2–119	2.05 × 10^−8^

## Data Availability

The data presented in this study are available on request from the corresponding author.
